# Correction: Reactions of [60]fullerene with alkynes promoted by OH^−^

**DOI:** 10.1039/d2ra90054h

**Published:** 2022-05-25

**Authors:** Wei-Wei Chang, Fa-Gui He, Alberto García-Peñas, Mehdihasan I. Shekh, Zong-Jun Li

**Affiliations:** Analysis & Testing Center, Shandong University of Technology Zibo 255049 China; State Key Laboratory of Electroanalytical Chemistry, Changchun Institute of Applied Chemistry, University of Chinese Academy of Sciences, Chinese Academy of Sciences Changchun 130022 China; State Key Laboratory of Catalysis, Dalian Institute of Chemical Physics, Chinese Academy of Sciences Dalian 116023 China hefg@dicp.ac.cn; Department of Materials Science and Engineering and Chemical Engineering, IAAB, University Carlos III of Madrid Madrid 28911 Spain; New Energy Materials Laboratory, College of Materials Science, Shenzhen University Shenzhen 518055 China

## Abstract

Correction for ‘Reactions of [60]fullerene with alkynes promoted by OH^−^’ by Wei-Wei Chang *et al.*, *RSC Adv.*, 2022, **12**, 14018–14021. https://doi.org/10.1039/D2RA01300B.

The authors regret that the name of one of the authors (Fa-Gui He) was shown incorrectly in the original article. The corrected author list is as shown above.

The Royal Society of Chemistry apologises for these errors and any consequent inconvenience to authors and readers.

## Supplementary Material

